# Astrocytomas IDH-mutant of posterior cranial fossa, clinical presentation, imaging features and onco-functional balance in surgical management

**DOI:** 10.1007/s10143-025-03436-x

**Published:** 2025-02-27

**Authors:** Matteo De Simone, Anis Choucha, Carlotta Ranalli, Giuseppina Pecoraro, Romain Appay, Oliver L. Chinot, Henry Dufour, Giorgio Iaconetta

**Affiliations:** 1 Department of Medicine, Surgery and Dentistry “Scuola Medica Salernitana”, University of Salerno, Via S. Allende, Baronissi, 84081 Italy; 2 Neuroanatomy Unit, BrainLab, Mercato San Severino, Salerno, 84085 Italy; 3 Department of Neurosurgery, APHM, UH Timone, Aix Marseille University, Marseille, 13005 France; 4 Laboratory of Biomechanics and Application, UMRT24, Gustave Eiffel University, Aix Marseille University, Marseille, 13005 France; 5 Department of Medicine and Surgery, Catholic University of Sacred Heart, Rome, Italy; 6 Service d’Anatomie Pathologique et de Neuropathologie, APHM, CHU Timone, Marseille, France; 7 Inst Neurophysiopathol, Aix-Marseille Univ, CNRS, INP, Marseille, France; 8 AP-HM, Service de Neuro-Oncologie, Aix-Marseille University, CHU Timone, Marseille, France

**Keywords:** IDH-mutant Astrocytomas, Posterior cranial fossa, WHO grade II-III-IV gliomas, Temozolomide, Onco-functional balance

## Abstract

IDH-mutant astrocytomas (AIDHmut) in the posterior cranial fossa (PCF) are rare and present substantial diagnostic and therapeutic challenges due to their location. We analyzed patients with PCF AIDHmut from our institutions, treated between December 2021 and September 2024. Additionally, we conducted a systematic literature review (from January 2021 to September 2024) using PubMed, Ovid MEDLINE, and Ovid EMBASE to identify cases of PCF AIDHmut. We identified a total of 19 cases, including one institutional case. Most patients were young adults, with a male predominance (15 males, 4 females). Tumors primarily originated from the brainstem (94.7%), with only one case involving the cerebellum. Clinical presentations frequently included cranial nerve deficits, with diplopia being the most common symptom (47.4%). Adjuvant radiotherapy (IMRT, DT 54 Gy/27 fractions, 78.9%) and chemotherapy (temozolomide, 68.4%) formed the mainstays of treatment. Tumor grading revealed 63.2% (12/19) were WHO grade 2, 21% (4/19) were WHO grade 3, and 15.8% (3/19) were grade 4. The mean follow-up period was 45 months. PCF AIDHmut are rare but pose significant treatment challenges due to their location and infiltrative nature. Multimodal treatment—comprising surgery, radiotherapy, and chemotherapy—is essential for achieving long-term disease control. Subtotal resection followed by adjuvant therapies provides a favorable balance between tumor control and functional preservation.

## Background

According to the fifth edition of the WHO Classification of Tumors of the Central Nervous System (WHO CNS-5, 2021), gliomas are divided into adult-onset or pediatric-onset. In the adult type, diffuse gliomas include astrocytoma (IDH-mutant), oligodendroglioma (IDH-mutant and 1p/19q-co-deleted), and glioblastoma (IDH-wildtype). These categories are further graded according to their malignancy potential, with astrocytomas ranging from WHO grades II to IV, oligodendroglioma ranging from WHO grades II to III, and glioblastomas being classified as WHO grade IV [1–3]. While glioblastomas make up approximately 55% of all gliomas, the remaining 45% comprise a mix of distinct histological subtypes [4, 5]. These new categories have been understudied, not only due to time constraints but also because much of the existing literature has yet to fully adopt the current nomenclature, hence the need for this paper.

Glial tumors of the posterior cranial fossa (PCF) are rare and primarily arise in the cerebellum or brainstem. Among these, brainstem gliomas (BSGs) are significantly more common in the pediatric population, accounting for up to 20% of all pediatric brain central nervous system (CNS) tumors. In contrast, brainstem gliomas are much rarer in adults, representing only 1–2% of adult brain gliomas [6]. The prognosis of BSGs varies significantly between pediatric and adult patients. Based on MRI characteristics, BSGs are categorized as either focal brainstem glioma (FBSGs), in 20% of cases, or diffuse pontine gliomas (DIPGs), in 80% of cases [7]. In adults, FBSGs are more frequent, allowing for more aggressive surgical treatment. However, in children, DIPGs are more common, making surgical resection more difficult, with radiotherapy often being the main palliative treatment, providing transient symptomatic improvement, and improves the overall survival (OS) duration by 3–6 months [8].

The PCF, the deepest compartment of the skull base, accommodates the cerebellum, brainstem, and associated cranial nerves (CNs III-XII). The most common PCF neoplasms are extra-axial tumors such as vestibular schwannomas (70–80%), followed by meningiomas (10–15%) and epidermoid cysts (5%) [9]. In the pediatric population, PCF tumors remain rare and frequently arise as secondary extensions from intrinsic neoplasms of the brainstem, cerebellar peduncle, or cerebellum, or from direct extensions from the fourth ventricle and lateral recess [6]. In children, the PCF may also harbor atypical tumors such as sarcomas, medulloblastomas, and ependymomas. Unlike in adult patients, clinicians should expect a higher likelihood of malignant histology in pediatric PCF tumors, especially in infants and young children, further highlighting the importance of thorough evaluation and timely intervention [7].

Unfortunately, because of the rarity and neuro-radiological features of PCF gliomas, such lesions are often misdiagnosed until confirmation by histological examination. The clinical relevance of the PCF tumors stems from the numerous types of lesions that can affect this region, often presenting with a variety of unclear symptoms, the most typical of these include tinnitus, hearing loss and balance disturbances such as dizziness [8].

In diffuse gliomas, a definitive cure remains elusive. The primary objective in managing these tumors is to achieve maximal resection, as the extent of resection is closely linked to patient prognosis [10, 11]. However, given the tumor’s location within the brain parenchyma, it is critical to avoid removing excessive amounts of normal brain tissue to maintain an optimal onco-functional balance. This balance is particularly challenging in the PCF, a region densely packed with vital neurovascular structures. Additionally, there is ongoing debate regarding the most effective adjuvant therapy for IDH-mutant gliomas, specifically whether temozolomide (TMZ) or the combination of procarbazine, lomustine, and vincristine (PCV) is superior [12–14].

To address these issues, we conducted a systematic review of AIDHmut gliomas in the PCF, with a focus on prognosis, adjuvant therapy, and the impact of specific mutations. In this study, we present also a case of AIDHmut located in the PCF to shed light on the clinical presentation, diagnosis, and management of these rare lesions.

## Materials and methods

### Literature review

#### Literature search

The systematic review was conducted according to the Preferred Reporting Items for Systematic Reviews and Meta-Analysis (PRISMA) guidelines [15]. A comprehensive literature search of the databases PubMed, Ovid MEDLINE, and Ovid EMBASE was conducted. The search was limited to the period from June 2021 to September 2024 to include only papers that incorporated the WHO V^th^ classification. The search was last updated on September 21, 2024. A combination of keyword searches was performed to generate a string. The search keywords, including “posterior cranial fossa”, “astrocytoma”, “grade II-III-IV”, “IDH-mutant’’, “cerebellar”, “brainstem” and “pons” were used in both “AND” and “OR” combinations. Studies were found using the Medical Subject Heading (MeSH) terms and Boolean operators. Other pertinent articles were identified through reference analysis of selected papers.

All studies were selected based on the following inclusion criteria: (1) case series or case reports, (2) lesions located in the PCF, (3) glial lesions of astrocytic nature, and (4) grade 2 to 4 astrocytomas according to the WHO V^th^ classification. Exclusion criteria were: (1) meta-analysis and literature reviews, and (2) articles not reporting sufficient data on the presentation, diagnosis, or management. The list of identified studies was imported into Endnote X9, and duplicates were removed. The coauthors (M.D.S. and A.C.) independently performed the screening of abstracts for eligibility. Discordance between authors was resolved by the consensus of senior authors (A.S. and G.I.).

#### Risk of bias assessment

The Newcastle-Ottawa Scale (NOS) was used to assess the quality of the included studies [16]. Quality assessment was performed by assessing the selection criteria, comparability of the study, and outcome assessment [17]. The ideal score was 9. Higher scores indicated better quality of studies. Studies receiving 7 or more points were considered high-quality studies. Two authors (A.C. and C.R.) performed the quality assessment independently. When discrepancies arose, papers were re-examined by a third author (M.D.S.).

#### Data extraction

For the institutional case and each study included in our systematic review, we extracted the following baseline information: number of patients, age, gender, and anamnesis of cancer familiarity or genetic disease. As for the age stratification, we adopted the recommended classes of the Adolescent and Young Adult Oncology Progress Review Group (AYAO PRG): children (0–15 y), young adults (16–39 y), adults (40–64 y), and elderly (≥ 65 y) [18]. Regarding the clinical presentation, we collected the following information: initial suspected diagnosis, pre-treatment Karnofsky Performance Status (KPS), histotype and molecular characteristics, tumor origin (i.e., pons, cerebellum), presenting symptoms and their duration, pre-treatment VII cranial nerve (CN) function [i.e., House-Brackmann (HB) grade]. As for the treatment, we extracted information regarding the surgical approach, type of resection [i.e., biopsy, sub-total resection (STR), near-total resection (NTR), gross-total resection (GTR)], and adjuvant therapy [i.e., chemotherapy (CHT) and/or radiotherapy (RT)]. For the outcomes, the post-treatment KPS and post-treatment VII CN function (HB grade), the time to recurrence (TTR), and the mean length of imaging and clinical follow-up were abstracted.

### Outcomes

Clinical presentation, diagnostic features, and management strategies were our primary objectives. Diagnostic features of AIDHmut of the PCF were examined, including clinical presentation, imaging findings, and differential diagnoses. The review also investigated the management strategies, including surgery, CHT, and/or RT. The secondary objectives were to assess the efficacy of these approaches based on clinical outcomes, survival, and recurrence rate.

### Statistical analysis

Descriptive statistics were reported including means and proportions. No formal statistical comparisons were performed due to small sample sizes and insufficient power to detect differences between groups.

## Results

### Illustrative case

**Fig. 1:**
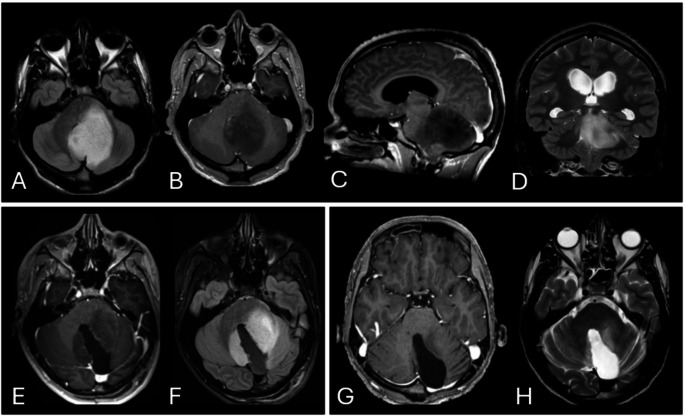
Preoperative (**A**, **B**, **C** & **D**), one-year postoperative (**E** & **F**), and two-year postoperative Brain MRI. (**G** & **H**). **A** preoperative axial T2-FLAIR MRI. **B** preoperative axial contrast-enhanced (CI) T1-weighted imaging (WI) MRI. **C** preoperative sagittal CI T1-WI MRI. **D** preoperative coronal T2-WI MRI. **E** one-year postoperative axial CI -T1-WI MRI. **F** one-year postoperative axial T2-FLAIR MRI. **G** two-year postoperative axial CI T1-WI MRI. **H** two-year postoperative axial T2-WI MRI

An 18-year-old male with no prior medical history presented with progressive intracranial hypertension and gait disorder over three weeks. His family history was notable for a cerebral rhabdomyosarcoma for her mother, treated 30 years earlier. Clinical examination showed cerebellar ataxia, adiadokokinesia predominantly on the left side, and subtle nystagmus. Ophthalmological assessment revealed mild bilateral papilledema. Brain MRI demonstrated an extensive left cerebellar lesion with pontine infiltration. The lesion appeared hypointense on T1-weighted sequences without tumoral enhancement after gadolinium injection – hyperintense on T2 and T2 FLAIR sequences (Fig. 1, A, B, C & D). There was associated triventricular hydrocephalus. The patient underwent urgent surgical intervention, first with third ventriculostomy followed by a subtotal resection of the cerebellar lesion a few days later. Histopathological and molecular analysis confirmed the diagnosis of WHO grade III anaplastic astrocytoma. Molecular findings included the absence of an IDH1 R132H mutation but revealed a minor IDH1 mutation (IDH1 R132G). There was a TP53 mutation, loss of nuclear ATRX expression, and unmethylated MGMT. The Ki-67 proliferation index was 50%. Genetic counseling ruled out Li-Fraumeni syndrome, the patient is does not have genetic condition. Following a multidisciplinary tumor board discussion, the patient underwent six cycles of chemotherapy (procarbazine, lomustine, and vincristine [PCV]) with adjuvant radiotherapy (60 Gy in 33 fractions) initiated between the third and fourth chemotherapy cycles. Post-treatment, the patient remained radiologically and clinically stable. PET-CT showed no hypermetabolism, and MRI revealed no concerning contrast enhancement (Fig. 1E, F, G & H). To this day, at 36 follow-up, the patient is working with accommodations for residual deficits, including exertional fatigue, noise intolerance, and mild residual cerebellar ataxia.

### Literature review

#### Literature search

**Fig. 2:**
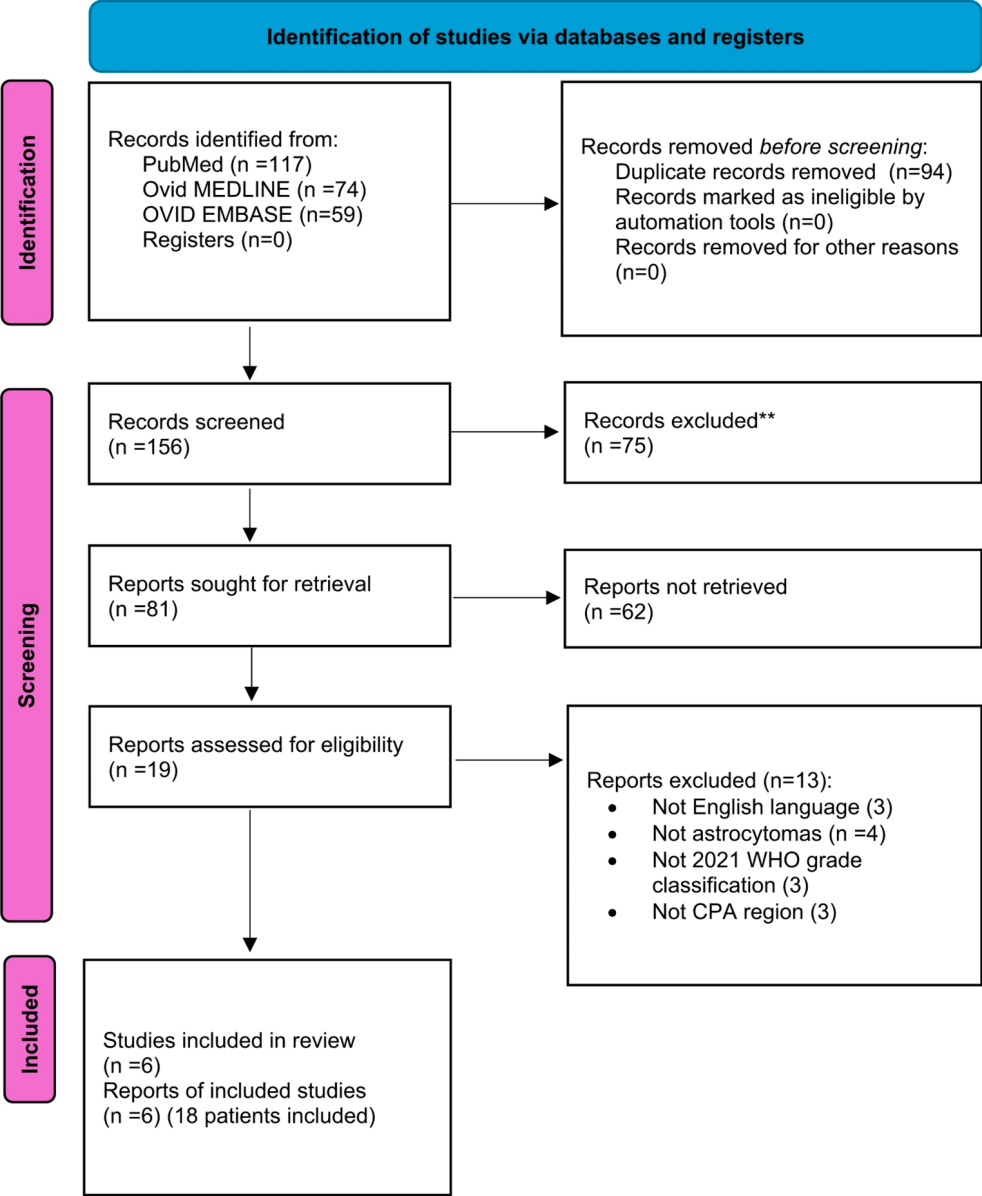
PRISMA flow diagram depicting the literature search process

A total of 156 papers were identified after duplicate removal. After title and abstract analysis, 19 articles were identified for full-text analysis. Eligibility was ascertained for 6 articles. The remaining 13 articles were excluded for reasons including (1) language other than English (3 articles), (2) glial lesions other than astrocytomas (4 articles), (3) lack of 2021 WHO grade classification (3 articles), (4) lesions not located in the CPA region (3 articles). Figure 2 shows the flow chart according to the PRISMA statement [19–24].

#### Pooled analysis

Including our Institutional case, a total of 19 patients harboring IDH-mutated astrocytoma in the posterior cranial fossa were studied in our systematic review. A male predilection (15:4) was observed. The median age was 31 years (range 17–52). According to the recommended age groups of the AYAO PRG [18], the series included 1 pediatric patient (17 years), 13 young adults (18–35 years: 18, 19, 21, 23, 24, 24, 27, 29, 32, 32, 33, 34, 35 years), 5 adults (36–49 years: 36, 38, 43, 49, 52 years). The median pre-treatment Karnofsky Performance Score (KPS) across the 19 patients, excluding the two with ‘not reported’ values, was calculated to be approximately 80.6.

Considering the histotype, the integrated diagnosis included astrocytoma, IDH-mutant, WHO grade 2 in 63.2% (12/19) of cases, WHO grade 3 in 21.1% (4/19) of cases, and WHO grade 4 in 15.8% (3/19) of cases. The IDH1 R132H mutation was the most frequent, occurring in 57.9% (11/19) of cases, followed by the IDH1 R132S mutation, which was found in 15.8% (3/19) of cases. The IDH1 R132G and IDH2 R172G mutations were each observed in 5.3% (1/19). In 15.8% (3/19) of cases, the mutation status was not reported (NR).

The origin of the tumor was most frequently in the brainstem, occurring in 94.7% (18/19) of cases, while only 1 case involved the cerebellum (1/19). The Ki-67 labeling index ranged from less than 1–50%, with an average of 10.9% (12/19). The index was not reported in 7 cases (7/19). Positivity for the TP53 mutation was observed in 73.7% (14/19) of cases, while it was not reported in 21.1% (4/19) of cases. Wilde-type TP53 was found in only one case (1/19). Loss of ATRX was observed in 26.3% (5/19) of cases, while in the remaining 73.7% (14/19) of cases, the status was not reported.

Analyzing the clinical presentation at diagnosis, diplopia was observed in 47.4% (9/19) of patients, while a broad category of other symptoms, including intracranial hypertension and impaired visual function, was present in 63.2% (12/19). Facial palsy or paralysis occurred in 36.8% (7/19), headache in 31.6% (6/19), and ataxia in 31.6% (6/19). Limb weakness or paralysis was noted in 26.3% (5/19), dizziness in 21.1% (4/19), and hearing loss, dysarthria, and hoarseness/dysphagia were each reported in 10.5% (2/19) of the cases. Diagnostic procedures varied, with open biopsy being the most common, performed in 26.3% (5/19) of cases.

**Fig. 3:**
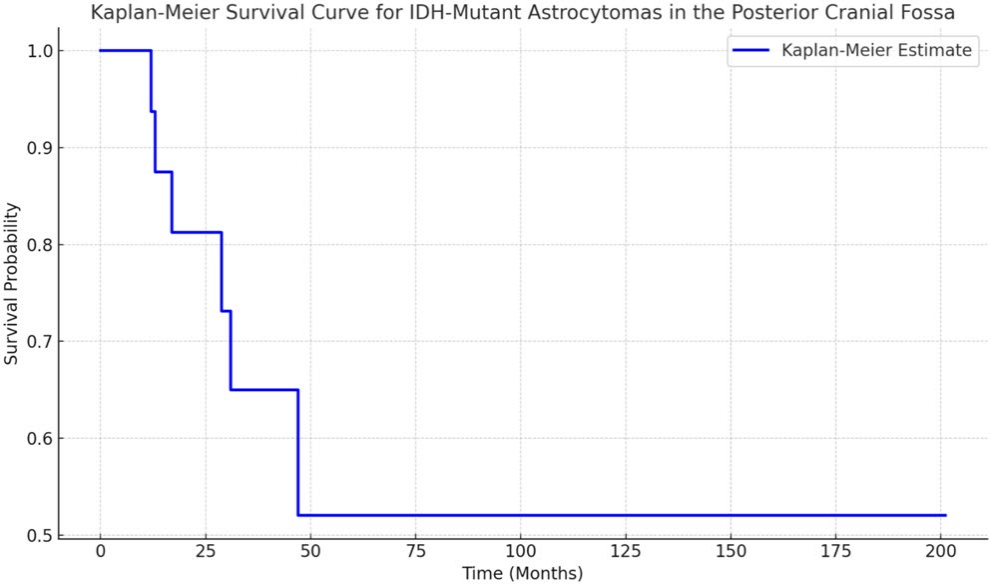
Kaplan-Meier survival curve for patients with IDH-mutant astrocytomas in the posterior cranial fossa

Navigation-guided open biopsy was used in 15.8% (3/19) of cases. Other techniques included brainstem excision and decompression procedures. The most common radiotherapy protocol utilized was IMRT (DT 54 Gy/27 fractions), applied in 78.9% (15/19) of cases. The most common chemotherapy agent used was temozolomide (TMZ), administered in 68.4% (13/19) of cases. The most frequent protocol involved TMZ (75 mg/m^2^) followed by continuous TMZ (150–200 mg/m^2^, day 1–5, every 4 weeks), for a total of 24 cycles, used in 15.8% (3/19) of cases. PCV (6 cycles) and a combination of TMZ, carboplatin, and vincristine were each used in 5.3% (1/19) of cases, for a total of 10.5% (2/19). Chemotherapy was not reported in 21.1% (4/19) of cases. The follow-up period was not reported in 21.1% (4/19) of cases, while it was available in 78.9% (15/19) of cases. Patients were followed up with a Gd-enhanced MRI. The median follow-up was 45 months (range 12–201 months) (Table 1; Fig. 3).

**Table 1:** Data collection of cases included

Author, Year	Age	Sex	Integrated diagnosis (or Histologic diagnosis)	KPS	IDH mutation type	TP53 Mutation	ATRX loss	Ki-67 labelling Index (%)	Tumor Location	Presenting symptoms	Diagnostic method (biopsy, STR, GTR)	SRS/RT (Gy/rads and No. of fractions if reported)	CHT (agent and No. of cycles if reported)	Follow-up (months)
Institutional case	18	M	Astrocytoma, IDH-mutant, WHO grade 3	90	IDH1 R132G	**+**	+	50%	Cerebellum	.Intracranial hypertension for 3 weeks .Cerebellar Ataxia .Gait Disturbance	Third ventriculostomy then STR	RT (1 cycle: 60gy 33fractions). The RT was performed between the 3rd and 4th PCV cycle)	PCV (6 cycles)	36
Oki et al., 2024	32	M	Astrocytoma, IDH-mutant, WHO grade 3	80	IDH1 R132H	-	NR	NR	Brainstem	.Left-sided facial tightness, headache; .Later developed diplopia; .Left facial palsy; .Ataxia	Two needle biopsies	IMRT (DT 54 Gy/27 fractions)	TMZ (150–200 mg/m^2^, day 1–5, every 4 weeks)	47 (death)
Oki et al., 2024	21	M	Astrocytoma, IDH-mutant, WHO grade 3	90	IDH2 R172G	+	NR	NR	Brainstem	.Left-sided deafness; .Two episodes of paralysis in the right upper extremity	.Open biopsy	IMRT (DT 54 Gy/27 fractions)	TMZ (75 mg/m^2^, followed by continuous TMZ (150–200 mg/m^2^, day 1–5, every 4 weeks), total of 24 cycles	NR
Oki et al., 2024	35	M	Astrocytoma, IDH-mutant, WHO grade 4	70	IDH1 R132H	**+**	**+**	NR	Brainstem	.Left facial palsy; .Hoarseness; .Diplopia; .Dysphagia; .Left ear hearing loss	.Autopsy diagnosis	IMRT (DT 54 Gy/27 fractions)	TMZ (75 mg/m^2^, followed by continuous TMZ (150–200 mg/m^2^, day 1–5, every 4 weeks), total of 24 cycles	28.8 (death)
Oki et al., 2024	36	M	Astrocytoma, IDH-mutant, WHO grade 4	90	IDH1 R132H	**+**	**+**	NR	Brainstem	.Headache; .Diplopia	Open Biopsy	IMRT (DT 54 Gy/27 fractions)	TMZ (75 mg/m^2^, followed by continuous TMZ (150–200 mg/m^2^, day 1–5, every 4 weeks), total of 24 cycles	31 (death)
Iwahashi et al. 2023	27	M	Astrocytoma, IDH-mutant, WHO grade 2	80*	IDH1 R132G	NR	NR	3%	Brainstem	NR	Komai stereotactic biopsy	IMRT (DT 54 Gy/27 fractions)	TMZ	63
Iwahashi et al. 2023	24	M	Astrocytoma, IDH-mutant, WHO grade 2	80*	IDH1 R132S	NR	NR	3%	Brainstem	NR	Navigation-guided open biopsy	IMRT (DT 54 Gy/27 fractions)	NR	64
Iwahashi et al. 2023	33	F	Astrocytoma, IDH-mutant, WHO grade 2	80*	IDH1 R132H	NR	NR	< 1%	Brainstem	NR	Navigation-guided open biopsy	IMRT (DT 54 Gy/27 fractions)	NR	26
Iwahashi et al. 2023	38	M	Astrocytoma, IDH-mutant, WHO grade 2	90*	IDH1 R132S	NR	NR	10%	Brainstem	NR	Navigation-guided open biopsy	IMRT (DT 54 Gy/27 fractions)	NR	201
Nagase et al., 2023	23	F	Astrocytoma, IDH-mutant, WHO grade 2	100	IDH1 R132H	**+**	**+**	NR	Brainstem	.Headache (lasting 6 months); .Impaired visual function; .Congested papillae	Stereotactic biopsy and foramen magnum decompression (FMD) (relieve obstructive hydrocephalus)	NR	NR	12
Zhou et al., 2022	34	M	Astrocytoma, IDH-mutant, WHO grade 2	70*	IDH1 R132H	**+**	**+**	5%	Brainstem	.Dizziness; .Diplopia; .Hearing loss in the right ear; .Dextral numbness;	GTR	IMRT (DT 54 Gy/27 fractions)	TMZ (120 mg)	NR
Zhou et al., 2022	49	M	Astrocytoma, IDH-mutant, WHO grade 2	60*	IDH1 R132H	**+**	NR	2%	Brainstem	.Limb Weakness; . Dysarthria; .Ataxia; .Dizziness	biopsy	IMRT (DT 54 Gy/27 fractions)	TMZ (75 mg/m^2^)	17
Zhou et al., 2022	29	M	Astrocytoma, IDH-mutant, WHO grade 2	70*	IDH1 R132H	**+**	NR	4%	Brainstem	.Episodic dizziness; .Diplopia; .Facial palsy	Robot-guided biopsy	IMRT (DT 54 Gy/27 fractions)	TMZ (75 mg/m^2^)	NR
Zhou et al., 2022	52	F	Astrocytoma, IDH-mutant, WHO grade 2	80*	NR	**+**	NR	7%	Brainstem	.Dizziness; .Ataxia: .Mild headache	.Brainstem glioma excision .Decompressive craniectomy .Extra-ventricular drainage	Conformal radiotherapy	NR	13
Zhou et al., 2022	32	M	Astrocytoma, IDH-mutant, WHO grade 2	80*	NR	**+**	NR	NR	Brainstem	.Weakness in the right limb	.Brainstem glioma excision, .Dura mater reparation .Cranioplasty	IMRT (DT 40 Gy/20 fractions)	TMZ (120 mg) with 6 cycles (340 mg)	28
Sano et al., 2021	43	M	Astrocytoma, IDH-mutant, WHO grade 4	NR	IDH1 R132H	**+**	NR	NR	Brainstem	.Diplopia (worsening over 24 months): .Right-sided sensory disturbance; .Left-sided Facial Paralysis	.Open biopsy (initially) . Subtotal tumor removal (following progression)	. IMRT (DT 54 Gy/27 fractions) .Stereotactic radiotherapy (36 Gy/9 fractions) after progression	.TMZ (75 mg/m^2^/day for 43 days) with radiotherapy, followed by 24 cycles of adjuvant TMZ (150–200 mg/m^2^/day)	NR
Chang et al., 2021	19	F	Astrocytoma, IDH-mutant, WHO grade 3	NR	IDH1 R132S	**+**	NR	20%*	Brainstem	.Left ptosis .Left facial palsy .Headache .Diplopia .Ataxia .Dysarthria	Open Biopsy	. IMRT (DT 54 Gy/27 fractions) . Focal Radiation	TMZ, carboplatin, vincristine	62
Chang et al., 2021	17	M	Astrocytoma, IDH-mutant, WHO grade 2	80*	IDH1 R132H	**+**	NR	< 5%*	Brainstem	.Left leg weakness .Headache .Diplopia .Ataxia .Left facial palsy	Open biopsy	IMRT (DT 54 Gy/27 fractions)	TMZ	36
Chang et al., 2021	24	M	Astrocytoma, IDH-mutant, WHO grade 2	80*	IDH1 R132H	**+**	NR	7%*	Brainstem	.Right facial palsy .Diplopia.	Open Biopsy	IMRT (DT 54 Gy/27 fractions)	TMZ	18

KPS = Karnofsky Performance Score; IDH = Isocitrate dehydrogenase; STR = subtotal resection; GTR = Gross total resection; CHT = chemotherapy; SRS/RT = Stereotactic Radiosurgery/Radiotherapy; Gy = Gray; DT = Dose Total; PCV = Procarbazine; TMZ = Temozolomide; IMRT = Intensity-Modulated Radiotherapy; NR = Not Reported

## Discussion

AIDHmut of the PCF are epidemiologically rare [9]. Only 19 cases of AIDHmut are described in the literature in this region. Patients of all age groups are included in this review (17–52 years). A predominance of distribution in the young adult group was observed. The medical history was negative for genetic syndromes in all cases. Their management is challenging, and the treatment strategies include surgery, CHT, and RT in different combinations. Based on our analysis of 19 cases, including the institutional case, several critical insights into clinical presentation, diagnosis, management, and outcomes can be drawn.

### Clinical presentation and diagnosis

All the patients reported had a pre-treatment KPS (reported or calculated) of 70 or more meaning that they had disabling symptoms but not such as to result in significant loss of independence. These findings are likely attributable to an early onset of symptoms resulting in rapid assessment by neuroradiological imaging. Early diagnosis of a disease can positively influences KPS in several ways. Early diagnosis often enables the initiation of timely and targeted treatments [25]. Treating a disease at an earlier stage can help reduce the impact on a patient’s overall function and preserve his or her quality of life. Besides, in cases where a disease is diagnosed early, there are generally fewer associated complications. The patient may have fewer severe symptoms or comorbidities, contributing to better functional capacity. Treatments such as CHT, RT, or surgery may be better tolerated when applied at an early stage of the disease. This may positively affect KPS, as the patient may maintain better function during and after treatment. In these types of tumors, preserving quality of life (QoL) is a clear priority. The surgeon’s role is becoming more like that of a judge, tasked with balancing the extent of tumor resection against the risk of neurological damage that may result from an aggressive approach. This requires evaluating the “onco-functional balance” on a case-by-case basis [26].

AIDHmut originating from pons tended to have a shorter duration of symptoms than those originating from the cerebellum [27]. The earliness of symptoms associated with pons AIDHmut compared with cerebellar AIDHmut could be attributed to several factors related to the anatomical location of the two brain regions and the functions they control. The pons is located in the brainstem, a critical region of the brain that regulates many vital functions, such as breathing, heart rate, and swallowing. Pons gliomas can involve and compress nerve pathways crucial for motor and sensory control. This can cause symptoms such as weakness, coordination problems, and sensory disturbances early on. It should also be considered that brain bridge gliomas, especially those with high aggressiveness, can progress rapidly and cause more pronounced mass effects than cerebellar gliomas. The more rapid growth and mass effects may lead to greater compression of neural structures, generating earlier symptoms. Radiologically, diffuse or nodular enhancement of the meninges can be observed, indicating infiltration by tumor cells. T1- and T2-weighted images may show areas of variable signal intensity in the meninges, which may be related to tumor infiltration, inflammation, or cerebrospinal fluid accumulation. Involved meninges may appear thickened, and irregular, and may show signs of irritation. It is common for PCF tumors, especially in pediatric patients, to present with intracranial hypertension caused by hydrocephalus [28–29]. Notably in the work of Won et al. brainstem compression (OR 5.4) was confirmed as an independent predictor of hydrocephalus in pediatrics. In general, the incidence of PFT-associated hydrocephalus at presentation is believed to be higher in children (70–90%) than in adults (10–21%) [30].

Despite the challenges associated with treating AIDHmut in the PCF, the median follow-up in this series was approximately 45 months, with no significant deterioration in KPS in most cases. Importantly, the institutional case demonstrated stable disease with no signs of recurrence at 36 months post-treatment. However, some cases in the literature reported recurrence or progression, emphasizing the importance of vigilant long-term follow-up, especially given the infiltrative nature of these tumors and their potential for malignant transformation. A large study with 20 years of follow-up on posterior cranial fossa tumors, published in 2020, reported that residual tumors were found in 40.2% of cases, with 10.9% of patients experiencing symptoms of disease progression. Tumor recurrence was observed in 14.3% of patients, while 6.0% developed severe disability. The overall mortality rate was 11.4% within the first year after surgery, rising to 18.9% in patients with malignant tumors. The 1-year event-free survival (EFS) was 66.0% for all patients. For those with malignant tumors, the 1-year EFS was 47.7%, compared to 87.7% for patients with benign tumors [31].

The pooled analysis further highlights the complexity of managing IDH-mutated astrocytomas in the PCF. The average Ki-67 labeling index (LI) was 10.9%, which may correlate with the overall favorable outcomes seen in this series. However, in cases with higher Ki-67 indices or more aggressive histopathological features, recurrence rates may be higher, necessitating more aggressive postoperative monitoring and potentially adjunctive therapies. Yang et al. demonstrated that morphological MRI (mMRI), SWI, DWI and SDC-PWI have the potential to predict Ki-67 LI and ATRX mutation status in AIDHmut. The combination of mMRI and SWI can improve the diagnostic performance for the prediction of Ki-67 LI and ATRX mutation status [32]. Regarding mutations, The IDH1R132H mutation was found in over 80% of supratentorial AIDHmut, while it was present in only 24% of infratentorial cases, where IDH1R132C/G mutations were more commonly observed [24, 26–33]. Mu et al., in a study of 55 patients, regarding mutations, found that patients with IDH1 R132H mutation had a longer recurrence free period (RFP) than the subgroup with IDH1 wild type (*p* <.01), but OS had no statistical difference between the two subgroups (*p* >.6). In addition, the IDH1 R132H mutation independently predicted a long RFP in patients with low grade remedial glioma (HR 1.073, 95% CI 0.151–0.775, *p* <.01) [34]. Regarding extent of resection (EOR), a recent retrospective study of 138 patients supplemented the molecular data with a survival analysis in pts with high-grade tumors. Patients with EOR ≥ 88% experienced 44% prolonged overall survival (OS) in multivariable analysis (hazard ratio: 0.56, *P* =.030). Patients with TP53 pathway alterations and EOR < 89% showed reduced OS compared with patients with TP53 pathway alterations and EOR > 89% (10.5 vs. 18.8 months; HR: 2.78, *P* =.013); however, EOR/RTV was not associated with OS in patients without TP53 pathway alterations. Meanwhile, in all patients with EOR < 88%, PTEN -altered had significantly worse OS than PTEN -wildtype (9.5 vs. 15.4 months; HR: 4.53, *P* <.001).

### Management and outcomes

Surgery is a useful strategy in many brain tumors. For some of these it is always the first choice, think of meningiomas. For the surgical management of posterior cranial fossa AIDHmut, the choice of approach is crucial for optimizing outcomes while minimizing risks. In the study by Bal et al. (2021) [35], eight patients with posterior incisural space lesions were treated using the suboccipital transtentorial (SOTT) approach. Gross or near-total resection (GTR or NTR) was achieved in 75% of cases, with no patient experiencing visual field deficits, and only one developing temporary trochlear nerve palsy. These results highlight the safety and efficacy of this approach in providing excellent access to deep-seated tumors, particularly when careful case selection is applied. Furthermore, Mahmoud et al. [36] reported on the surgical management of exophytic brainstem gliomas, achieving near-total excision in 87% of cases. They found that the telovelar approach, used in 17 out of 23 patients, allowed for significant tumor removal with minimal complications.

Similarly, Rady et al. (2024) [37] analyzed pediatric brainstem and peduncular low-grade gliomas (LGGs), demonstrating that gross total resection was achieved in 40% of cases, with near-total resection in 34%. The study also found a clear difference in outcomes based on the extent of resection: the 3-year progression-free survival rate was 100% for GTR cases, compared to 88.2% for NTR, highlighting the importance of maximal safe resection in improving long-term outcomes.

Cinibulak et al. (2024) [38] compared the transmastoid infralabyrinthine approach (TI-A) techniques for jugular fossa pathologies and found that the short-rerouting technique significantly increased the exposed area (169.1 mm^2^ vs. 151.0 mm^2^) and surgical freedom (19,650 mm^2^ vs. 17,233 mm^2^) compared to the non-rerouting technique. However, tailored steps to the non-rerouting approach provided comparable access, making it a valuable option for select cases, particularly for class C1, C2, and C3 lesions.

Regarding extent of resection (EOR), a recent retrospective study of 138 patients supplemented the molecular data with a survival analysis in pts with high-grade tumors [39]. Patients with EOR ≥ 88% experienced 44% prolonged overall survival (OS) in multivariable analysis (hazard ratio: 0.56, *P* =.030). Patients with TP53 pathway alterations and EOR < 89% showed reduced OS compared with patients with TP53 pathway alterations and EOR > 89% (10.5 vs. 18.8 months; HR: 2.78, *P* =.013); however, EOR/RTV was not associated with OS in patients without TP53 pathway alterations. Meanwhile, in all patients with EOR < 88%, PTEN -altered had significantly worse OS than PTEN -wildtype (9.5 vs. 15.4 months; HR: 4.53, *P* <.001).

In an article, predating the latest classification, the authors related the extent of resection to survival in a retrospective court of 74 patients with low-grade gliomas [40]. Stratifying by IDH status, showed that higher EOR independently prolonged malignant progression-free survival (MPFS) and OS for wild type IDH patients (hazard ratio [HR] = 0.002 [95% confidence interval {CI} 0.000-0.074] and HR = 0.001 [95% CI 0.00-0.108], respectively), but not for IDH-mutant patients (HR = 0.84 [95% CI 0.17–4.13] and HR = 2.99 [95% CI 0.15–61.66], respectively). The role of EOR on OS in grade 3 tumors was recently analyzed in a retrospective court of 246 patients, and it was shown that extended resection benefits only a portion of patients with WHO grade 3 IDH-mutant gliomas; in particular, stratifying for age, no survival benefit from total resection was found in patients with astrocytoma older than 45 years [41].

Those interesting findings makes it clear that the biology of these tumors is complex, and the role of surgery is not sufficient, which is why adjuvant treatment modalities are increasingly emerging and finding a venue. In fact, in this regard, TMZ-based chemoradiotherapy is associated with a survival benefit in patients with grade 2 AIDHmut. Chemoradiotherapy can be deferred until the time of progression in younger patients undergoing extensive resection, while early treatment should be recommended in high-risk patients [42]. These debates represent research areas of keen interest; beyond this is the discourse of molecular understanding that is also increasingly prominent in brain tumors, which is why immunotherapeutic strategies may be useful. The latter have shown potential in various cancer types, but their efficacy in gliomas has been limited by tumor heterogeneity and an immunologically “cold” microenvironment. However, promising results from recent studies exploring vaccines, T-cell therapies, and IDH-mutant inhibitors offer hope for improved treatment outcomes in AIDHmut gliomas [4, 43, 44].

Considering this evidence, it is imperative to plot prospective studies aimed at understanding what treatment is most appropriate and what the surgical timing is, and it will also be essential to determine what the goal of surgery will be in these cases.

### Limitations

This paper was primarily based on retrospective studies and thus has limitations inherent to retrospective studies. Because of the small number of studies responding to the inclusion criteria, our paper was limited to descriptive statistics. Nonetheless, to the best of our knowledge, we reported 19 cases of IDH-mutant astrocytoma in the PCF region, and we provide a comprehensive synopsis of the presentation, clinical challenges, and management of these tumors. The systematic review also included a risk of bias assessment using the Newcastle-Ottawa Scale (NOS). The NOS allowed for the evaluation of the quality of the included studies based on selection criteria, comparability of the study, and outcome assessment.

## Conclusions

AIDHmut tumors of the PCF are rare but present significant clinical and surgical challenges. Recent updates in classification have highlighted the importance of multimodal treatment, including subtotal resection followed by adjuvant chemotherapy and radiotherapy, which appear to offer favorable outcomes while minimizing functional deterioration. Although surgical intervention is often limited, earlier diagnosis allows patients to benefit more from adjuvant therapies. The delicate balance between maximizing tumor resection and preserving neurological function—known as the ‘onco-functional balance’—is crucial in guiding treatment decisions on an individual basis. The results of this study highlight that the nosological entity *AIDHmut* when located in the PCF, requires specialized diagnostic and management approaches. Our analysis identified a male predominance (15:4) with a median age of 31 years, spanning pediatric to adult groups. The tumor was predominantly located in the brainstem (94.7%), with only one case involving the cerebellum. Most cases were classified as WHO grade 2 (63.2%), followed by grade 3 (21.1%) and grade 4 (15.8%). Molecularly, the IDH1 R132H mutation was the most common (57.9%), while TP53 positivity was observed in 73.7% of cases, and ATRX loss in 26.3%. Treatment primarily included IMRT-based radiotherapy (78.9%) and temozolomide chemotherapy (68.4%), underscoring a targeted approach in managing this rare tumor. This finding opens the door to potential advancements in the implementation of the WHO classification. Further research, involving larger patient cohorts and longer follow-up, is necessary to refine therapeutic strategies and improve overall patient outcomes.

## Data Availability

Data is provided within the manuscript or supplementary information files.

## Declarations

### Submission

This manuscript is original and has not been submitted elsewhere in part or in whole.

## References

[1] Ostrom QT, Price M, Neff C et al (2023) CBTRUS statistical report: primary brain and other central nervous system tumors diagnosed in the united States in 2016–2020. Neurooncology 25(Supplement4):iv1–iv99. 10.1093/neuonc/noad149 10.1093/neuonc/noad149 PMC10550277 37793125

[2] Gesaka SR, Okemwa PM, Philip Maseghe Mwachaka (2024) Histological types of brain tumors diagnosed at the Kenyatta National Hospital between 2016 and 2019: a retrospective study. Discover Oncol 15(1). 10.1007/s12672-024-00893-6 10.1007/s12672-024-00893-6 PMC10874916 38368566

[3] Louis DN, Perry A, Wesseling P et al (2021) The 2021 WHO classification of tumors of the central nervous system: a summary. Neuro Oncol 23(8):1231–1251. 10.1093/neuonc/noab106 34185076 10.1093/neuonc/noab106 PMC8328013

[4] De Simone M, Conti V, Palermo G, De Maria L, Iaconetta G (2024) Advancements in glioma care: focus on emerging neurosurgical techniques. Biomedicines 12:8. 10.3390/biomedicines12010008 10.3390/biomedicines12010008 PMC10813759 38275370

[5] Mair MJ, Geurts M, van den Bent MJ, Berghoff AS (2021) A basic review on systemic treatment options in WHO grade II-III gliomas. Cancer Treat Rev 92:102124. 10.1016/j.ctrv.2020.102124 33227622 10.1016/j.ctrv.2020.102124

[6] Reyes-Botero G, Mokhtari K, Martin‐Duverneuil N, Delattre J, Laigle‐Donadey F (2012) Adult brainstem gliomas. Oncologist 17(3):388–397. 10.1634/theoncologist.2011-0335 22382458 10.1634/theoncologist.2011-0335 PMC3316925

[7] Dine N et al (2022 Jun) Childhood brainstem gliomas: A non-aggressive management. Interdiscip Neurosurg 28:101488. doaj.org/article/21fb236a7d024e00833cfa73197dd2fd. Accessed 23 Sept. 2024

[8] Vanan MI, Eisenstat DD (2015 Oct) DIPG in children – What can we learn from the past? Front Oncol 21;5. 10.3389/fonc.2015.00237 10.3389/fonc.2015.00237 PMC4617108 26557503

[9] Bray HN, Sappington JM (2022) A review of posterior Fossa lesions. Mo Med 119(6):553–558. https://www.ncbi.nlm.nih.gov/pmc/articles/PMC9762221/36588644 PMC9762221

[10] Duffau H, Mandonnet E (2013b) The onco-functional balance in surgery for diffuse low-grade glioma: integrating the extent of resection with quality of life. Acta Neurochir 155(6):951–957. 10.1007/s00701-013-1653-9 23447053 10.1007/s00701-013-1653-9

[11] Still MEH, Roux A, Huberfeld G, Bauchet L, Baron M, Fontaine D, Blonski M, Mandonnet E, Guillevin R, Guyotat J, Taillandier L, Capelle L, Duffau H, Pallud J (2018) Extent of resection and residual tumor thresholds for postoperative total seizure freedom in epileptic adult patients harboring a supratentorial diffuse Low-Grade glioma. Neurosurgery 85(2):E332–E340. 10.1093/neuros/nyy481 10.1093/neuros/nyy481 30395304

[12] Dono A, Ballester LY, Primdahl D, Esquenazi Y, Bhatia A (2021) IDH-Mutant Low-grade glioma: advances in molecular diagnosis, management, and future directions. Curr Oncol Rep 23(2):20 Published 2021 Jan 25. 10.1007/s11912-020-01006-6 33492489 10.1007/s11912-020-01006-6

[13] Esteyrie V, Dehais C, Martin E, Carpentier C, Uro-Coste E, Figarella-Branger D, Bronniman C, Pouessel D, Ciron DL, Ducray F, Moyal EC, Network P (2021) Radiotherapy plus procarbazine, lomustine, and vincristine versus radiotherapy plus Temozolomide for IDH-Mutant anaplastic astrocytoma: A retrospective multicenter analysis of the French POLA cohort. Oncologist 26(5):e838–e846. 10.1002/onco.13701 33524191 10.1002/onco.13701 PMC8100568

[14] Tesileanu CMS, Sanson M, Wick W, Brandes AA, Clement PM, Erridge SC, Vogelbaum MA, Nowak AK, Baurain J-F, Mason WP, Wheeler H, Chinot OL, Gill S, Griffin M, Rogers L, Taal W, Rudà R, Weller M, McBain C, van Linde ME (2022) Temozolomide and radiotherapy versus radiotherapy alone in patients with glioblastoma, IDH-wildtype: post hoc analysis of the EORTC randomized phase III CATNON trial. Clin Cancer Research: Official J Am Association Cancer Res 28(12):2527–2535. 10.1158/1078-0432.CCR-21-4283 10.1158/1078-0432.CCR-21-4283 PMC9297529 35275197

[15] Page MJ, McKenzie JE, Bossuyt PM et al (2021) The PRISMA 2020 statement: an updated guideline for reporting systematic reviews. BMJ (Clinical Res ed) 372. 10.1136/BMJ.N71 10.1136/bmj.n71 PMC8005924 33782057

[16] Ottawa Hospital Research Institute. Accessed December 8 (2020) http://www.ohri.ca/programs/clinical_epidemiology/oxford.asp

[17] Luchini C, Stubbs B, Solmi M, Veronese N (2017) Assessing the quality of studies in meta-analyses: advantages and limitations of the Newcastle Ottawa scale. World J Meta-Analysis 5(4):80–84. 10.13105/wjma.v5.i4.80

[18] Albritton K, Anderson B, Nichols C (2021) Closing the Gap: research and care imperatives for adolescents and young adults with Cancer. Report of the Adolescent and Young Adult Oncology Progress Review Group. https://ascopubs.org/doi/10.1200/OP.21.00223

[19] Nagase T, Ishida J, Sasada S, Sasaki T, Otani Y, Yabuno S, Fujii K, Uneda A, Yasuhara T, Date I (2023) IDH-mutant Astrocytoma arising in the brainstem with symptom improvement by foramen magnum decompression: A case report. NMC Case Rep J 10(0):75–80. 10.2176/jns-nmc.2022-0159 37065877 10.2176/jns-nmc.2022-0159 PMC10101703

[20] Sano K, Matsuda K, Kawanami K, Kanemura Y, Ohe R, Sonoda Y (2021) Malignant progression of an IDH mutant brainstem glioma in adult. NMC Case Rep J 8(1):301–307. 10.2176/nmccrj.cr.2020-0151 35079479 10.2176/nmccrj.cr.2020-0151 PMC8769401

[21] Zhou J, Lai M, Ni Y, Li S, Zhen J, Du F, Zhang X, Song C, Cai L (2022) Case report: clinicopathological and genetic features of IDH-Mutant brainstem glioma in adults: report of five cases. Pathol Oncol Res 28. 10.3389/pore.2022.1610408 10.3389/pore.2022.1610408 PMC9385964 35991838

[22] Oki S, Ishi Y, Sawaya R, Okamoto M, Motegi H, Tanei Z, Tsuda M, Mori T, Nishioka K, Kanno-Okada H, Aoyama H, Tanaka S, Yamaguchi S, Fujimura M (2024) Clinical outcome, radiological findings, and genetic features of IDH-mutant brainstem glioma in adults. Acta Neurochir 166(1). 10.1007/s00701-024-06154-3 10.1007/s00701-024-06154-3 38864949

[23] Iwahashi H, Nagashima H, Tanaka K, Uno T, Hashiguchi M, Maeyama M, Somiya Y, Komatsu M, Hirose T, Itoh T, Sasaki R, Sasayama T (2023) 2-Hydroxyglutarate magnetic resonance spectroscopy in adult brainstem glioma. J Neurosurg 139(2):355–362. 10.3171/2022.12.jns221954 36708540 10.3171/2022.12.JNS221954

[24] Chang EK, Smith-Cohn MA, Tamrazi B, Ji J, Krieger M, Holdhoff M, Eberhart CG, Margol AS, Cotter JA (2021) IDH‐mutant brainstem gliomas in adolescent and young adult patients: report of three cases and review of the literature. Brain Pathol 31(4). 10.1111/bpa.12959 10.1111/bpa.12959 PMC8412065 33960568

[25] Grant R, Dowswell T, Tomlinson E, Brennan PM, Walter FM, Ben-Shlomo Y, Hunt DW, Bulbeck H, Kernohan A, Robinson T, Lawrie TA (2020) Interventions to reduce the time to diagnosis of brain tumours. Cochrane Database Syst Rev 9(9):CD013564. 10.1002/14651858.CD013564.pub2 32901926 10.1002/14651858.CD013564.pub2 PMC8082957

[26] Duffau H, Mandonnet E (2013) The onco-functional balance in surgery for diffuse low-grade glioma: integrating the extent of resection with quality of life. Acta Neurochir (Wien) 155(6):951–957. 10.1007/s00701-013-1653-9 23447053 10.1007/s00701-013-1653-9

[27] Leibetseder A, Leitner J, Mair MJ, Meckel S, Hainfellner JA, Aichholzer M, Widhalm G, Dieckmann K, Weis S, Furtner J, von Oertzen T, Preusser M, Pichler J, Berghoff AS (2022) Prognostic factors in adult brainstem glioma: a tertiary care center analysis and review of the literature. J Neurol 269(3):1574–1590. 10.1007/s00415-021-10725-0 34342680 10.1007/s00415-021-10725-0 PMC8857120

[28] Due-Tønnessen BJ, Helseth E (2007) Management of hydrocephalus in children with posterior fossa tumors: role of tumor surgery. Pediatr Neurosurg 43(2):92–96. 10.1159/000098379 17337918 10.1159/000098379

[29] Muthukumar N (2021 Nov–Dec) Hydrocephalus associated with posterior fossa tumors: How to manage effectively? Neurology India 69(Suppl 2):S342-S349. 10.4103/0028-3886.332260 10.4103/0028-3886.332260 35102986

[30] Won SY, Dubinski D, Behmanesh B et al (2020) Management of hydrocephalus after resection of posterior fossa lesions in pediatric and adult patients-predictors for development of hydrocephalus. Neurosurg Rev 43(4):1143–1150. 10.1007/s10143-019-01139-8 31286305 10.1007/s10143-019-01139-8

[31] Bhat AR, Wani MA, Kirmani AR (2020) Histopathological pattern and outcome of posterior Fossa tumors in children and Adults - A 20-Year experience. Asian J Neurosurg 15(2):285–292. 10.4103/ajns.AJNS_120_19 32656120 10.4103/ajns.AJNS_120_19 PMC7335140

[32] Yang X, Hu C, Xing Z et al (2023) Prediction of Ki-67 labeling index, ATRX Tation, and MGMT promoter methylation status in IDH-mutant Astrocytoma by morphological MRI, SWI, DWI, and DSC-PWI. Eur Radiol 33(10):7003–7014. 10.1007/s00330-023-09695-w 37133522 10.1007/s00330-023-09695-w

[33] Banan R, Stichel D, Bleck A, Hong B, Lehmann U, Suwala A, Reinhardt A, Schrimpf D, Buslei R, Stadelmann C, Ehlert K, Prinz M, Acker T, Schittenhelm J, Kaul D, Schweizer L, Capper D, Harter PN, Etminan N, Reuss DE (2020) Infratentorial IDH-mutant Astrocytoma is a distinct subtype. Acta Neuropathol 140(4):569–581. 10.1007/s00401-020-02194-y 32776277 10.1007/s00401-020-02194-y

[34] Mu L, Xu W, Li Q, Ge H, Bao H, Xia S, Ji J, Jiang J, Song Y, Gao Q (2017) IDH1 R132H mutation is accompanied with malignant progression of paired Primary-Recurrent astrocytic tumours. J Cancer 8(14):2704–2712. 10.7150/jca.20665 28928859 10.7150/jca.20665 PMC5604202

[35] Bal J, Fairhead RJ, Matloob S, Shapey J, Romani R, Gavin C, Shoakazemi A, Pollock J (2023) The use of the suboccipital transtentorial approach to the posterior inferior incisural space. Cureus 15(10);e47705. 10.7759/cureus.47705 10.7759/cureus.47705 PMC10674890 38021782

[36] Mahmoud AT, Enayet A, Alselisly AMA (2021) Surgical considerations for maximal safe resection of exophytic brainstem glioma in the pediatric age group. Surg Neurol Int 12:310. 10.25259/sni_318_2021 34345451 10.25259/SNI_318_2021 PMC8326137

[37] Rady MR, Enayet AE, Refaat A et al (2022) Management and outcome of pediatric brainstem and cerebellar peduncular low-grade gliomas: a retrospective analysis of 62 cases. Childs Nerv Syst 38(3):565–575. 10.1007/s00381-021-05405-3 34787716 10.1007/s00381-021-05405-3

[38] Cinibulak Z, Poggenborg J, Schliwa S, Al-Afif S, Ostovar N, Krauss JK, Nakamura M (2024) Assessing the feasibility of the transmastoid infralabyrinthine approach without decompression of the jugular bulb to the extradural part of the petrous apex and petroclival junction prior to surgery. Acta Neurochir 166(1):151. 10.1007/s00701-024-06044-8 38530445 10.1007/s00701-024-06044-8 PMC10965636

[39] Dono A, Zhu P, Takayasu T et al (2024) Extent of resection thresholds in molecular subgroups of newly diagnosed isocitrate Dehydrogenase-Wildtype glioblastoma. Neurosurgery 95(4):932–940. 10.1227/neu.0000000000002964 38687046 10.1227/neu.0000000000002964 PMC12245224

[40] Patel T, Bander ED, Venn RA et al (2018) The role of extent of resection in IDH1 Wild-Type or mutant Low-Grade gliomas. Neurosurgery 82(6):808–814. 10.1093/neuros/nyx265 28945860 10.1093/neuros/nyx265 PMC7571505

[41] Hou Z, Hu J, Liu X et al (2023) Decision system for extent of resection in WHO grade 3 gliomas: a Chinese glioma genome atlas database analysis. J Neurooncol 164(2):461–471. 10.1007/s11060-023-04420-5 37668945 10.1007/s11060-023-04420-5

[42] Minniti G, Paolini S, Antonelli M, Gianno F, Tini P, Lanzetta G, Arcella A, De Pietro R, Giraffa M, Capone L, Romano A, Bozzao A, Esposito V (2023) Long-term treatment outcomes of temozolomide-based chemoradiation in patients with adult-type diffuse IDH-mutant grade 2 Astrocytoma. J Neurooncol 164(2):331–339. 10.1007/s11060-023-04418-z 37665475 10.1007/s11060-023-04418-z PMC10522719

[43] Gallus M, Kwok D, Lakshmanachetty S, Yamamichi A, Okada H (2023) Immunotherapy approaches in Isocitrate-Dehydrogenase-Mutant Low-Grade glioma. Cancers 15(14):3726. 10.3390/cancers15143726 37509387 10.3390/cancers15143726 PMC10378701

[44] Nagle VL, Henry KE, Hertz CAJ et al (2021) Imaging Tumor-Infiltrating lymphocytes in brain tumors with [^64^Cu]Cu-NOTA-anti-CD8 PET. Clin Cancer Res 27(7):1958–1966. 10.1158/1078-0432.CCR-20-3243 33495310 10.1158/1078-0432.CCR-20-3243 PMC8026513

